# 

**Published:** 1909-05

**Authors:** 


### EDITORIAL DEPARTMENT.

THE ANNUAL MEETING of the Texas State Medical Association at Galveston, May 11, 12 and 13, is being looked forward to with unusual interest. A very large attendance is expected, and a splendid program, both for the meeting and for social pleasures, has been prepared and published in the Association's journal for April. Galveston, always attractive, will be unusually so in the delightful month of May, and a cordial welcome by her splendid people awaits us. Visitors will be delightfully entertained with true Southern hospitality, and it is hoped members will be accompanied by their wives, daughters and sweethearts.

